# Concentration of Access to Information and Communication Technologies in the Municipalities of the Brazilian Legal Amazon

**DOI:** 10.1371/journal.pone.0152655

**Published:** 2016-04-01

**Authors:** Silvana Rossy de Brito, Aleksandra do Socorro da Silva, Adejard Gaia Cruz, Maurílio de Abreu Monteiro, Nandamudi Lankalapalli Vijaykumar, Marcelino Silva da Silva, João Crisóstomo Weyl Albuquerque Costa, Carlos Renato Lisboa Francês

**Affiliations:** 1 Cyberspace Institute, Federal Rural University of Amazon, Pará, Brazil; 2 Institute of Applied Social Sciences, Federal University of Pará, Pará, Brazil; 3 Amazon Higher Studies Nucleus, Federal University of Pará, Pará, Brazil; 4 Special Technologies Center, National Institute for Space Research, São Paulo, Brazil; 5 Institute of Technology, Federal University of Pará, Pará, Brazil; University of Rijeka, CROATIA

## Abstract

This study fills demand for data on access and use of information and communication technologies (ICT) in the Brazilian legal Amazon, a region of localities with identical economic, political, and social problems. We use the 2010 Brazilian Demographic Census to compile data on urban and rural households (i) with computers and Internet access, (ii) with mobile phones, and (iii) with fixed phones. To compare the concentration of access to ICT in the municipalities of the Brazilian Amazon with other regions of Brazil, we use a concentration index to quantify the concentration of households in the following classes: with computers and Internet access, with mobile phones, with fixed phones, and no access. These data are analyzed along with municipal indicators on income, education, electricity, and population size. The results show that for urban households, the average concentration in the municipalities of the Amazon for computers and Internet access and for fixed phones is lower than in other regions of the country; meanwhile, that for no access and mobile phones is higher than in any other region. For rural households, the average concentration in the municipalities of the Amazon for computers and Internet access, mobile phones, and fixed phones is lower than in any other region of the country; meanwhile, that for no access is higher than in any other region. In addition, the study shows that education and income are determinants of inequality in accessing ICT in Brazilian municipalities and that the existence of electricity in rural households is directly associated with the ownership of ICT resources.

## Introduction

Access to information is a fundamental right in democratic societies. This is established via the principles and provisions of the Universal Human Rights Declaration [1] as well as the Millennium Objectives [2]. For some researchers, access to information and communications technologies (ICT) is essential for economic development [3–4], social equality [5–6], improvement in health care [6–8] and education systems [6, 9], innovation in the financial sector [10], improvement of social services and governance [11–13], changes in public policies of countries [14], and expansion of democratic participation [15–16].

ICT include computers, the Internet, computer software, peripheral equipment, and mobile and fixed-line telephones that allow access to information and communication between entities, such as individuals, organizations, and countries. The existence of entire communities without access to ICT is still a reality in much of the world and in Brazil. According to the International Telecommunications Union (ITU) [17], 4.3 billion people are still not online, and 90 per cent of them live in the developing world. The result is that these communities are sidelined from the benefits of economic globalization and do not belong to the global information society. This is the situation for many slave-descendant (*quilombola*) and indigenous communities that live in riverside and isolated rural areas of the Brazilian Legal Amazon.

Location is one of the main factors of the digital divide in many countries. For example, Chinese citizens who reside in urban areas are much more likely to have access to the Internet than those who live in rural areas; there is an Internet penetration rate of 62.8% in urban areas of China, but only 28.8% in rural areas of China [18]. There are still rural provinces in China where the divide with urban areas is very prominent, such as Sichuan province [19]. The urban–rural gap also occurs in the ownership of phones (fixed or mobile). For example, according to the latest population and housing census carried out in India in 2011, 82% of Indian urban households have access to a telephone compared with 54% of rural households [20].

ICT statistics are receiving increasing attention owing to the impact these technologies have on economies and societies in general [20]. These statistics are proposed to measure both domestic characteristics (in a given country or region) [18, 21–26] and international characteristics (gaps between countries or continents) [20, 27–28]. For example, based on the ICT Development Index from the ITU [20] that measures the global digital divide between countries, Novo-Corti and Barreiro-Gen [22] proposed a regional index to evaluate the comparative impact on different Spanish regions, grouping the regions through cluster analysis and calculating a discriminate regional index. To analyze factors associated with availability and utilization of ICT in US states, Pick et al. [23] proposed in a study an exploratory conceptual model that shows association of ICT utilization with social capital, education, societal openness, urbanization, and ethnicity.

According Ribeiro et al. [25], socioeconomic level and socio-spatial location are determinant criteria for computer ownership and Internet access. For the authors, households with higher amounts of material resources (income) and intangible resources (education) are most likely to access opportunities offered by the Internet. The association between the level of digital inclusion of individuals and their income has been the subject of several studies [17, 24–26, 29], which show that the lower the income of the household and/or individual is, the lower is the level of digital inclusion of individuals. In addition, the association between education and digital inclusion has been the subject of studies that show that the lower the educational level of the individual is, the lower is the level of skills for the use of ICT [17, 24, 25, 29].

Brazil is a country of continental dimensions, with large inequalities in access to ICT. For example, São Caetano do Sul in São Paulo state, has the highest rate in the country of households with computers with Internet (74%) compared to Aroeiras in Paraíba state, which has almost no access [24]. Inequalities are greatest when comparing urban and rural areas. According to data from the National Household Sample Survey 2013 (*Pesquisa Nacional por Amostra de Domicílios; PNAD*) [30], which is a sample survey that provides indicators at the state level, 80% of individuals of 10 years or older in urban areas of Brazil had mobile phones for personal use, against only 47.9% in rural areas. In the North region, this inequality is more significant: 77.3% of individuals in urban areas of the North had mobile phone, against 34.3% of people in rural areas of the North. Despite Brazil’s economic and social growth in past years, severe inequalities in access to ICT in urban and rural areas reflect the challenges of achieving universal access. ICT could make a particularly significant impact in these poor and rural areas of country, although ICT performance is better in urban areas, where there is more favorable access to ICT infrastructure, usage, and skills [17].

In Brazil, there is a lack of studies on the concentration of access to ICT performed at the level of municipalities, taking as a reference the Brazilian Legal Amazon. Such studies could support planning of specific public policies for the Amazon—a region that brings together counties with similar characteristics, due to economic, political, and social problems of the region. Our research attempts to fill this gap.

The objective of this study is to analyze the concentration of access to ICT in the municipalities of the Brazilian Legal Amazon region compared to other regions of the country. The analysis of concentration is performed on 2010 Demographic Census data, using household data about computers with Internet access, mobile phones, and fixed phones. We attained this objective by applying the concentration index. The results are analyzed along with municipal indicators on income, education, existence of electricity, and population size.

The remainder of this paper is organized as follows. In the “Materials and Methods” section, we describe the conceptual model, study area and population, data sources, and methods used in this research. In the “Results and Discussion” section, we present a detailed description of the results by applying the described methods. Finally, in the “Conclusion,” we present the conclusions from this study.

## Materials and Methods

### Conceptual Model

A high level of ICT access is viewed by the United Nations as a basic human right [1] that must be assured on an equal basis to all citizens in a globalized society. However, despite advances in the quality and availability of ICT, the level of access or skills varies widely among citizens. According to the ITU [17], high service costs and a lack of ICT skills have been identified as major barriers to increased ICT use.

**Fig 1:**
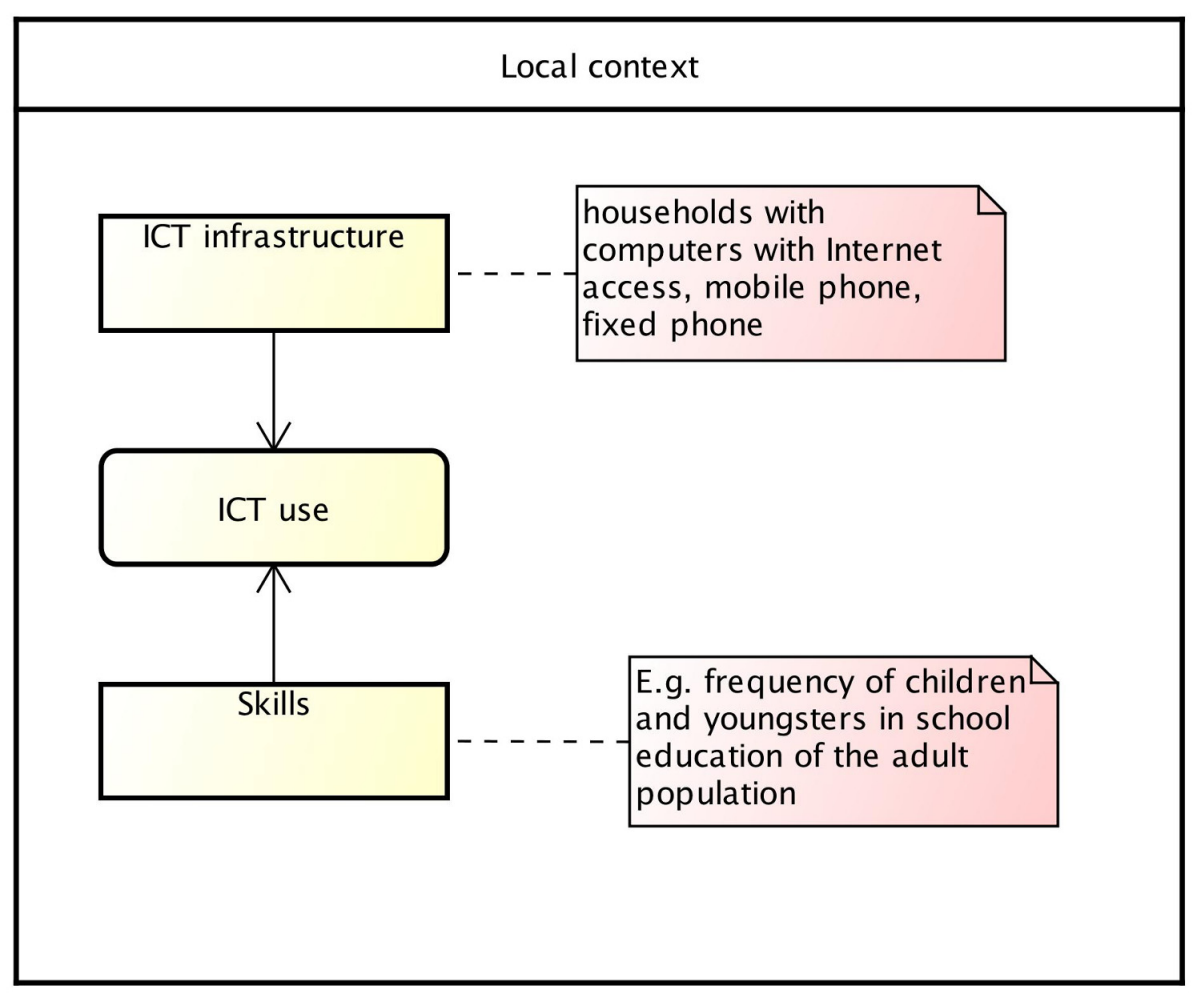
Relationship between ICT Access, ICT Use, and Skills.

Based on ICT Development Index from the ITU [17], which measures the global digital divide between countries using indicators of ICT access, ICT use, and ICT skills, we developed the conceptual model from which we identify the variables of our study. In this model, the intensity and effectiveness of ICT use depends on the level of network infrastructure and access as well as skills in use, as shown in Fig 1. For this, we adopted the following guidelines proposed by the ITU [17]: (i) ICT infrastructure can be measured by Internet bandwidth per user, mobile and fixed telephone subscriptions, number of computers, and computers with Internet access within households; (ii) Skills can be measured by adult literacy rates, secondary gross enrolment ratios, and tertiary gross enrolment ratios; (iii) ICT use can be measured by percentage of individuals using the Internet, fixed (wired)-broadband subscriptions per 100 inhabitants, and wireless-broadband subscriptions per 100 inhabitants.

This study focuses on the concentration of ICT access in Brazilian municipalities. For this, we selected representative variables of ICT infrastructure in households from the Brazilian Demographic Census for each municipality. For us, local context represents the municipalities that have specific characteristics (i.e., rural/urban, income and education educators, existence of electricity, and population size).

### Study Area and Population

**Fig 2:**
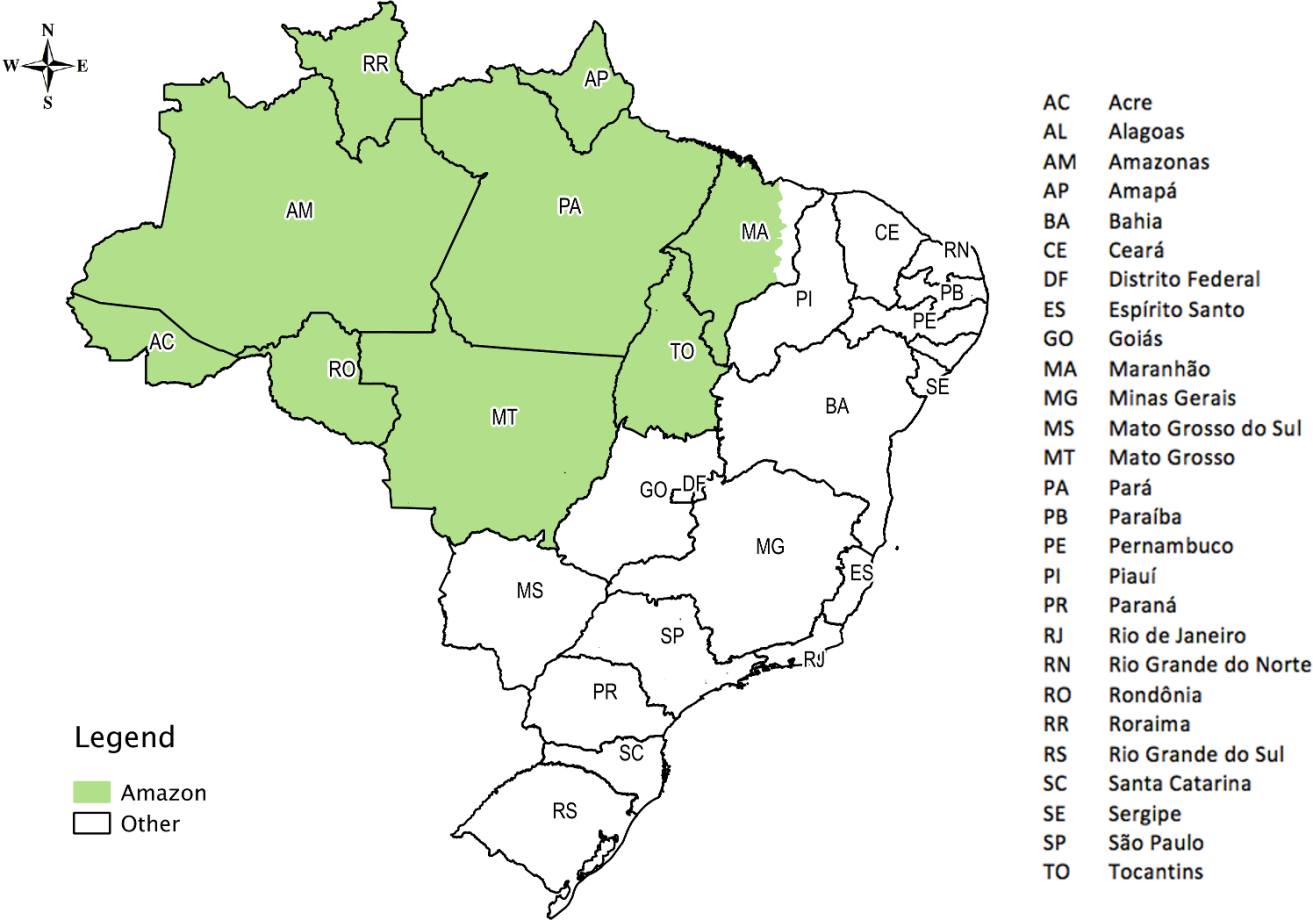
Brazil with 27 federative units in the Amazon and other regions.

Brazil is divided into five geographic regions (North, Northeast, South, Southeast, and Central-West), 27 federative units (FU) comprising 26 states and one federal district, and 5,507 municipalities, 756 of which are located in the Brazilian Legal Amazon, using the administrative division of the country in the year 2000 as a reference. The Brazilian Legal Amazon is a political-administrative division that comprises all the states of the North region (Acre, Amapá, Amazonas, Pará, Rondônia, Roraima, and Tocantins), the Mato Grosso state, in the Central-West, and part of the Maranhão state, in the Northeast (Fig 2) (*Instituto Brasileiro de Geografia e Estatística; IBGE*; http://www.ibge.gov.br).

In this study, we use data from the Household Survey of the Demographic Census 2010, performed on 57,320,474 households by the IBGE. We analyzed ICT infrastructure in urban and rural households (owning a computer with Internet, mobile phone, and fixed phone) and municipal indicators of education, income, population size, and electricity. The classification of urban and rural areas is according to current Brazilian legislation during the census.

Henceforth, the term “Brazilian Legal Amazon” will be referred to simply as “Amazon.”

### Data Sources

Based on variables used by ITU [17] for measuring ICT access as well as our conceptual model, we used the public data of the Demographic Census in 2010 of the IBGE (http://loja.ibge.gov.br/populacao/universo.html) to collect information about each household in 5,507 municipalities, with respect to: (i) designated area of households (urban or rural); (ii) existence of computers with Internet access; (iii) existence of mobile phones; and (iv) existence of fixed phones. The public microdata were obtained directly from the IBGE as an anonymized set of records. This study used Brazilian municipalities as spatial reference units by distinguishing those belonging to the Amazon from those located in any other region of Brazil. We used the latest census data from 2010 because the question “owning a personal computer and Internet access” was added only in this census and the next census will be in the year 2020. The selected variables from the census and the possible answers are presented in Table 1.

**Table 1:** Selected Variables from the 2010 Census about the Existence of Personal Computers with Internet Access, and Mobile or Fixed Phones.

Variable (Census)	Description	Possible answers
*V1006*	Type of household	1 –Urban / 2 –Rural
*V0220*	Existence of households with computers with Internet access	1 –Yes / 2 –No
*V0217*	Existence of mobile phones	1 –Yes / 2 –No
*V0218*	Existence of fixed phones	1 –Yes / 2 –No

With respect to municipalities, we used the classification of population size and the distribution of the number of Amazon municipalities and other regions from the IBGE (*IBGE*; http://www.sidra.ibge.gov.br/bda/tabela/listabl.asp?c=1378&z=cd&o=7) to define the variable *size_population* (Table 2). We used the variable *local* to identify whether a municipality belongs to the Amazon or another region of the country.

**Table 2:** Distribution of Municipalities of the Amazon and other Regions of the Country.

Class of municipality(*size_population*)	Description	Number of municipalities		Total
		Amazon	Other regions	
Metropolis	more than 900,000 inhabitants	3	15	18
Large	from 100,001 to 900,000 inhabitants	28	236	264
Medium	from 50,001 to 100,000 inhabitants	58	266	324
Small	up to 50,000 inhabitants	667	4,234	4901
Total		756	4,751	5507

In addition, we collected education and income indicators for 2010 for each municipality from the platform Atlas of Human Development in Brazil (*Atlas do Desenvolvimento Humano no Brasil*; http://www.atlasbrasil.org.br). These indicators are components of the Municipal Human Development Index (MHDI) and are classified by the Institute for Applied Economic Research (*Instituto de Pesquisa Econômica Aplicada*) [31], as shown by the categories and ranges of values in Table 3.

**Table 3:** Income and Education Indicators for Brazilian Municipalities.

Indicator	Description	Category	Range of values
MHDI income	Represented by the variable *mhdi_income*, it corresponds to the component “income” of the MHDI. It is measured by the municipal income per capita, that is, the average income of residents of a given municipality.	very low	0.000 ≤ MHDI income ≤ 0.499
		low	0.500 ≤ MHDI income ≤ 0.599
		medium	0.600 ≤ MHDI income ≤ 0.699
		high	0.700 ≤ MHDI income ≤ 0.799
		very high	0.800 ≤ MHDI income ≤ 1.000
MHDI education	Represented by the variable *mhdi_education*, it corresponds to the component “education” of the MHDI. This is based on the geometrical average of the frequency sub-index of children and youngsters in school (weight 2/3) and the education sub-index of the adult population (weight 1/3).	very low	0.000 ≤ MHDI education ≤ 0.499
		low	0.500 ≤ MHDI education ≤ 0.599
		medium	0.600 ≤ MHDI education ≤ 0.699
		high	0.700 ≤ MHDI education ≤ 0.799
		very high	0.800 ≤ MHDI education ≤ 1.000

The values for MHDI Income and MHDI Education are represented by numbers ranging between “0” and “1”; the closer the number is to “1,” the higher is the component (income or education) of human development of a municipality. These value ranges are defined and categorized by the Institute for Applied Economic Research (*Instituto de Pesquisa Econômica Aplicada*) [31].

With respect to the existence of electricity network in Brazilian households—represented in this study by the variable *electricity*—we obtained the percentage for each municipality from the Demographic Census via the IBGE’s automatic recovery system (IBGE; http://www.sidra.ibge.gov.br/bda/tabela/listabl.asp?z=t&o=1&i=P&e=l&c=1395).

### Methods

**Fig 3:**
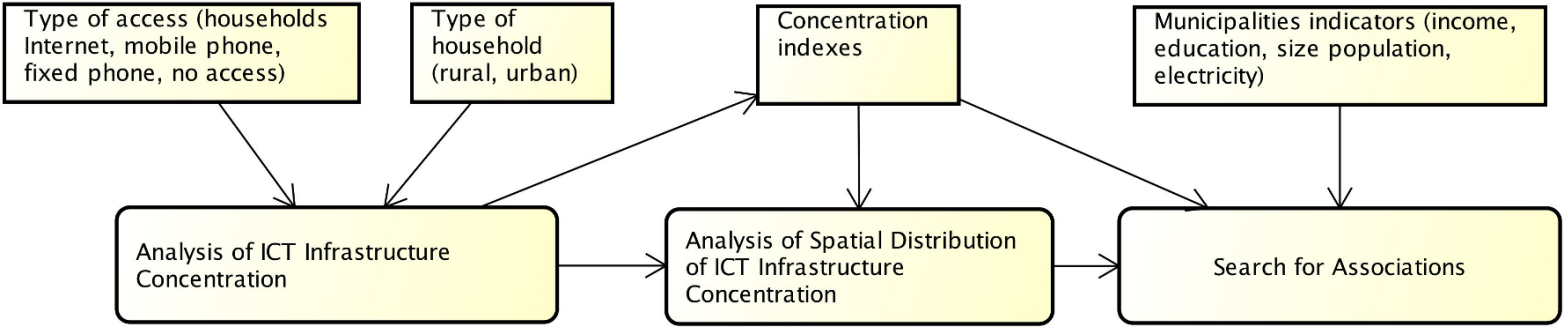
Approach to Analysis Concentration of ICT access.

Our approach (Fig 3) is to (i) analyze the ICT infrastructure concentration in Brazilian municipalities; (ii) analyze the spatial distribution of ICT infrastructure concentration in urban and rural households in Brazilian municipalities; and (iii) search for associations between parameters describing the ownership of ICT resources in urban and rural households with indicators for income, education, population size, and the existence of electricity in municipalities. In the first step of analyzing ICT infrastructure concentration, we produced and analyzed concentration indexes according to the type of access (internet access, mobile phone, and fixed phone) and type of households (rural or urban). The concentration indexes were used in the second step of analyzing spatial distribution of ICT infrastructure concentration to produce maps that show the concentration of Internet access, mobile phone, and fixed phone in the municipalities. Finally, in the third step of searching for associations, we investigated associations between municipal indicators of income, education, population size, existence of electricity, and concentration indexes.

In the analysis of ICT infrastructure concentration, for type of access, we defined eight classes (Table 4), which determine the characteristics of households with respect to type of household (urban or rural) and access type from the four variables selected in the census (Table 1). Each access type represents a variable in this study: *internet_access*, *mobile_phone*, *fixed_phone*, and *no_access*.

**Table 4:** Classes and Variables.

Class	Type of household	Variable for type of access	Description	Variables from Census 2010^*^			
				*V1006*	*V0220*	*V0217*	*V0218*
1	urban	*internet_access*	Urban households with computers with Internet access	1	1	X	X
2	urban	*mobile_phone*	Urban households with mobile phones and without computers with Internet access.	1	2	1	X
3	urban	*fixed_phone*	Urban households with fixed phones, without computers with Internet access, and without mobile phones.	1	2	2	1
4	urban	*no_access*	Urban households without computers with Internet access and without phones (fixed or mobile).	1	2	2	2
5	rural	*internet_access*	Rural households with computers with Internet access	2	1	X	X
6	rural	*mobile_phone*	Rural households with mobile phones and without computers with Internet access.	2	2	1	X
7	rural	*fixed_phone*	Rural households with fixed phones, without computers with Internet access, and without mobile phones.	2	2	2	1
8	rural	*no_access*	Rural households without computers with Internet access and without phones (fixed or mobile).	2	2	2	2

* 1 –Yes; 2 –No; X–Any value

In sequence, we used a normalized concentration index (CI) to quantify the concentration of each class (*internet_access*, *mobile_phone*, *fixed_phone*, and *no_access*) in each of the territorial units analyzed, that is, municipalities. The CI is an index that aims to identify local productive agglomerations and its processing follows the methodology developed by Crocco et al. [32]. Local productive agglomerations or local productive clusters can be defined as sectoral and spatial concentrations of firms [33]. We extended this concept to reference the spatial concentration of households according to the ownership of ICT resources (microcomputers with Internet access, mobile phones, and fixed phones). This index is composed from three other indexes:

(1) Location Quotient (*LQ*), as defined in Eq 1, is an index that helps to determine whether a municipality has a particular specialization in a specific class:

LQij=EijEjEiE (1)

(2) Herfindahl–Hirschman modified (*HHm*), as defined in Eq 2, is a modification of the Hirschman–Herfindahl Index, used by Crocco et al. [32] to capture the weight of the class in the municipality:

$$HHm_{ij}=\frac{E_{ij}}{E_{i}}-\frac{E_{j}}{E}$$ (2)

(3) Relative participation (RP), as defined in Eq 3, which measures the relative participation of the class in the municipality in relation to a region:

$$RP_{ij}=\frac{E_{ij}}{E_{i}}$$ (3)

where:

*E*_*ij*_ is the occurrence of class *i* in municipality *j*;

*E*_*j*_ is the total occurrence in municipality *j*;

*E*_*i*_ is the occurrence of class *i* considering the region under study;

*E* is the total occurrence considering all classes and the region under study.

The combination of these indexes has the purpose of capturing the following: (i) how concentrated a particular class *i* is in a municipality *j* compared to the nation, by *LQ*; (ii) the weight of a class *i* in a municipality *j* in the nation in relation to the weight of all classes of the municipality as a total of all the nation's classes, by *HHm*; and (iii) the importance of the class *i* in a municipality *j* in relation to the total of such a class in the nation, by *RP*.

Based on these indexes, the CI is calculated as in Eq 4:

$$CI_{ij}=\theta_{1}LQ_{ij}+\theta_{2}HHm_{ij}+\theta_{3}RP_{ij}$$ (4)

where *θ*_1_, *θ*_2_, and *θ*_3_ are the weights of each index for each particular class.

To calculate the weights, we applied principal component analysis, which is a multivariate statistical technique that analyzes a data table representing observations described by several dependent variables, which are generally inter-correlated [34]. The goal of principal component analysis is to extract the important information from the table in order to represent it as a set of new orthogonal variables called principal components.

CI was processed for each class (*i*) and for each municipality (*j*) of the country. Thus, the values of *internet_access*, *mobile_phone*, *fixed_phone*, and *no_access* of urban and rural households are compared by considering if the municipality belongs to the Amazon region or any other region. In addition, we process the average CI for Brazil, the Amazon, and the other regions of the country, identifying the municipalities with the highest value in each class. Since this index has no maximum or minimum limits, we consider municipalities with a high degree of concentration, in other words, those that have above-average CIs in the analyzed region, as suggested by Crocco et al. [32].

For the “Analysis of Spatial Distribution of ICT Infrastructure Concentration”, we produce maps that show the concentration of urban and rural households in *internet_access* using the frequency method to define the intervals for the CI. In addition, we produce maps that combine different scenarios: (i) concentration in *no_access* above the average of country; (ii) concentration in *mobile_phone* above the average of country and below the average in *internet_access*, excluding the occurrences in (i); and (iii) concentration in *internet_access* above the average of country, also excluding the occurrences in (i). The georeferenced cartographic database of the Brazilian states and municipalities is freely available online in shapefile format by the Brazilian Institute of Geography and Statistics (http://downloads.ibge.gov.br/downloads_geociencias.htm.). We used Qgis software version 2.6.0-Brighton (http://qgis.org), a free and open-source geographic information system, to elaborate all the maps in this study.

In “Search for associations”, we used the RP, which indicates the contribution of a particular municipality (*j*) to the *i* class, to analyze the classes according to income and education indicators and according to population size of the municipalities. As the RP represents percentage participation, it is natural that the most populated municipalities tend to contribute more in all classes because they are the municipalities with the highest number of households. Along this direction, from the results of the RP according to population size, we calculated the average CI for urban and rural households in relation to the population size of these municipalities, comparing the Amazon with other regions of the country.

In sequence, based on the attributes of municipalities (population size, income and education indicators, and existence of electricity) and variables representing ownership of ICT resources in the households (*internet_access*, *mobile_phone*, *and fixed_phone*), we advanced to applying a Bayesian network technique, also referred to as a causal network or graphic model of probabilistic dependence, in order to analyze the associations between these variables. Bayesian networks are models that encode probabilistic relationships among variables that represent a certain domain. These models include both a qualitative and quantitative structure. The qualitative structure represents dependencies between nodes (variables), while the quantitative structure represents the conditional probabilities of these nodes. The idea is evaluate the nodes in probabilistic terms [35–36] and to provide a compact and easy-to-use representation of the probabilistic information from the data. We used the K2 heuristic search algorithm [37] to find the most probable Bayesian network structure within the search space. This network structure is an effective way to communicate dependencies among the domain variables.

The ownership of ICT resources was processed as the average between CI for *internet_access*, *mobile_phone*, and *fixed_phone* for each municipality according to type of household and is represented by the variable *ict*. Thus, the ownership of ICT resources was synthesized into one value. In sequence, we created two datasets as input files for the Bayesian analysis—one for each type of household, containing the variables *mhdi_income*, *mhdi_education*, *size_population*, *local*, *ict*, *electricity*. Once the network was established, the posterior distribution of the parameters was estimated by statistical inference.

### Ethics Statement

This study is based on secondary data, and all presented information is in the public domain. No variables allowed identification of individuals or households. Thus, approval of the study by an Ethical Review Board was not necessary.

## Results and Discussion

### Analysis of ICT Infrastructure Concentration

**Fig 4:**
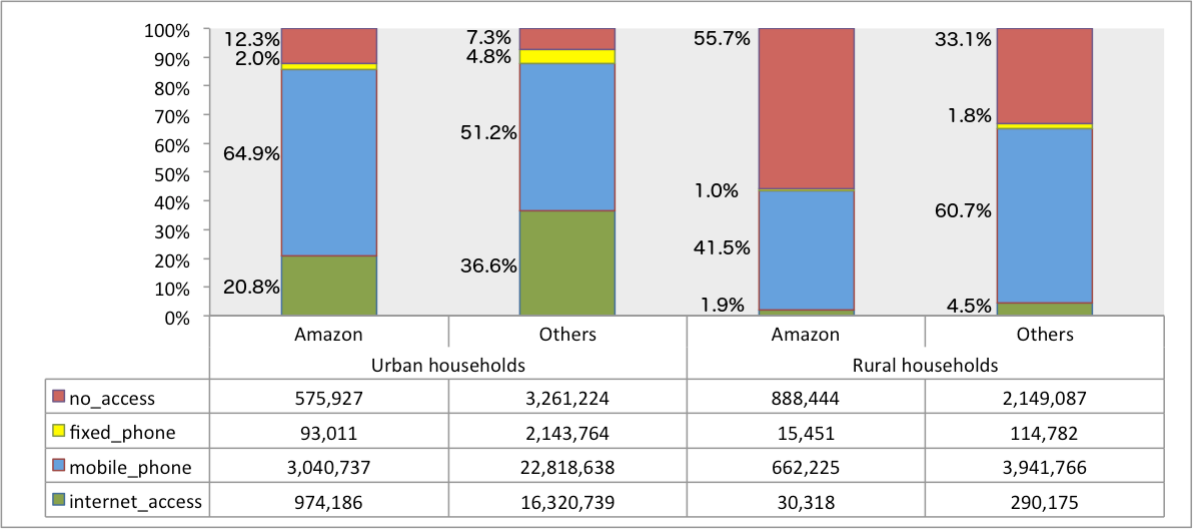
Urban and Rural Households, According to Type of Access for the Amazon and Other Regions.

With regard to *internet_access* and *fixed_phone* in urban households, the Amazon has the lowest percentage (20.8% and 2%, respectively) compared to other regions of the country (36.6% and 4.8%, respectively). For rural households, the Amazon has lower percentages of *internet_access* (1.9%), *mobile_phone* (41.5%), and *fixed_phone* (1%) compared to other regions of the country (4.5%, 60.7%, and 1.8%, respectively); for *no_access*, the percentage in the Amazon is even more pronounced: 55.7% of rural households without access to ICT, against 33.1% in the other regions. Among the hypotheses that can be posited for the worse indicators in the Amazon relative to other regions are the low income, low educational level, and poor telecommunications infrastructure in these municipalities. Fig 4 shows the numbers and percentages of households according to type of access in the Amazon and other regions of the country.

However, in urban households, the Amazon has the highest percentage of mobile phone ownership in relation to others (64.9% vs. 51.2%); this can be justified by the low percentage of fixed phones in the households, as result of technical difficulties of installing infrastructure for fixed telephony services in the region. It is important to note that the highest percentage of mobile phone ownership indicates only the existence of the resource and not the penetration rate or number of mobile phones per inhabitant.

We applied principal component analysis and found weights *θ*_1_, *θ*_2_, and *θ*_3_ for *LQ*, *HHm*, and *RP*, respectively (Table 5).

**Table 5:** Weights for *LQ*, *HHm*, and *RP*.

Class	Type of household	Variable for type of access	*θ*_1_	*θ*_2_	*θ*_3_
1	urban	*internet_access*	0.3321	0.3368	0.3310
2	urban	*mobile_phone*	0.3278	0.3310	0.3411
3	urban	*fixed_phone*	0.3318	0.3239	0.3443
4	urban	*no_access*	0.3339	0.3164	0.3497
5	rural	*internet_access*	0.3327	0.3272	0.3402
6	rural	*mobile_phone*	0.3557	0.3049	0.3394
7	rural	*fixed_phone*	0.3326	0.3273	0.3401
8	rural	*no_access*	0.3443	0.3111	0.3445

The weights were used to calculate the CI for each municipality in each analyzed class. For urban households (S1 File), sorted in decreasing order by the degree of concentration in *internet_access*, there was a predominance of municipalities in the South and Southeast regions of Brazil. In fact, the first municipality in the Amazon to appear in the descending order of *internet_access* was Cuiaba, the capital of Mato Grosso state, in 179^th^ position; in this municipality, 44% of urban households had computers with Internet access. Even among the 500 municipalities with the highest CI in *internet_access*, only 2 were located in the Amazon: Cuiabá and Primavera do Leste, both in Mato Grosso state.

On the other hand, for the municipalities with the lowest CI value for urban households with regard to *no_access*, there was a predominance of municipalities of the Amazon and of the Northeast (S1 File). Among the top 50 municipalities with the highest concentration in this class, 50% were in the Amazon, including the municipality with the highest CI score (2.972), Itamarati, located in Amazonas state. For the bottom 50 of the expanded list, that is, with lesser degrees of *no_access*, we found no municipalities located in the Amazon.

We performed the same process for rural households. Among the top 50 municipalities with the highest concentration in *internet_access*, all belonged to South and Southeast regions (S2 File). The Amazon municipality with the highest CI is Campos de Júlio (2.424) in Mato Grosso state, with a ranking of 54; only 28% of its rural households had computers with Internet access. Even when we considered the first 500 municipalities with the highest CI in *internet_access*, only 13 were located in the Amazon. There was a predominance of municipalities of the Amazon and the Northeast with the highest CI for *no_access* (S2 File). When we considered the top 50 municipalities, only 12 were not located in the Amazon.

Table 6 shows the average CI for the four classes among urban households for Brazil, the Amazon, and other regions of the country. For *no_access*, the average CI of Amazon municipalities was higher than the average CI for the other regions. In addition, Table 6 shows the municipalities with the highest concentration in all classes. Itamarati and Água Azul do Norte, both located in the Amazon, had the highest concentration for *mobile_phone* and *no_access*, respectively. Itamarati (AM) is an isolated municipality in the Amazon that is served only with fluvial transport to other municipalities. On the other hand, Água Azul do Norte (PA), with the highest concentration in *mobile_phone*, is adjacent to the exploration of mineral reserves on Carajás, a region of high economic potential; the high concentration of *mobile_phone* can be justified by the low concentration of *fixed_phone* as result of unequal distribution of telephone services in the country until 2010. Sao Caetano do Sul (SP), a municipality located in the country’s Southeast region, has the highest concentration of *internet_access* for urban households; this was the municipality with the highest MHDI of Brazil in 2010, as well as the lowest concentration of *no_access*. Iomerê (SC), in the South, had a high concentration of *fixed_phone* and a low concentration of *mobile_phone*.

**Table 6:** Average CI for Urban Household Classes and Municipalities with the Highest CI in Each Class.

Region	State	Municipality	Urban households			
			*internet_access*	*mobile_phone*	*fixed_phone*	*no_access*
Brazil	-	(average)	0.184	0.377	0.300	0.683
Amazon	-	(average)	0.100	0.412	0.145	0.914
Other regions	-	(average)	0.198	0.372	0.324	0.647
Southeast	São Paulo	São Caetano do Sul	**0.650**	0.139	0.553	0.066
North (Amazon)	Pará	Água Azul do Norte	0.050	**0.548**	0.000	0.293
South	Santa Catarina	Iomerê	0.374	0.186	**1.942**	0.176
North (Amazon)	Amazonas	Itamarati	0.006	0.170	0.194	**2.972**

Highest values are listed in bold.

Table 7 shows the municipalities with the highest concentration of rural households in all the classes as well as the average CI for Brazil, the Amazon, and other regions. For *internet_access*, the municipality with the highest CI was Xangri-lá (6.720), located in Rio Grande do Sul state, in which 80% of its rural households had computers with Internet access. In Ivatuba, in Paraná state, which had the second highest CI in this class (4.426), 53% of rural households had computers with Internet access.

**Table 7:** Average CI for Rural Household Classes and Municipalities with the Highest CI in Each Class.

Region	State	Municipality	Rural households			
			*internet_access*	*mobile_phone*	*fixed_phone*	*no_access*
Brazil	Brazil	(average)	0.412	0.388	0.390	0.286
Amazon	Amazon	(average)	0.145	0.280	0.188	0.482
Other regions	Other regions	(average)	0.455	0.405	0.422	0.254
South	Rio Grande do Sul	Xangri-lá	**6.720**	0.125	0.000	0.000
Southeast	Minas Gerais	Caxambu	0.000	**0.625**	0.000	0.000
Central-West	Goiás	Santa Bárbara de Goiás	0.000	**0.625**	0.000	0.000
Southeast	Minas Gerais	Caxambu	0.000	**0.625**	0.000	0.000
Southeast	São Paulo	Américo Brasiliense	0.000	**0.625**	0.000	0.000
Southeast	São Paulo	Ilhabela	0.000	**0.625**	0.000	0.000
Southeast	São Paulo	Dobrada	0.000	**0.625**	0.000	0.000
Southeast	São Paulo	Santa Gertrudes	0.000	**0.625**	0.000	0.000
Southeast	Minas Gerais	Timóteo	0.000	**0.625**	0.000	0.000
South	Rio Grande do Sul	Esteio	0.000	**0.625**	0.000	0.000
Southeast	São Paulo	Paulínia	0.000	**0.625**	0.000	0.000
South	Rio Grande do Sul	Imbé	0.000	**0.625**	0.000	0.000
South	Santa Catarina	Princesa	0.257	0.246	**7.707**	0.186
North (Amazon)	Acre	Santa Rosa do Purus	0.000	0.002	0.000	**0.915**

Highest values are listed in bold.

For the highest CI in *mobile_phone* and *fixed_phone* (Table 7), there were no municipalities located in the Amazon; on the other hand, the highest CI in *no_access* belongs to the Amazon. For *no_access*, like for urban households, the average CI indicates a higher concentration of households with no access to ICT in the Amazon. One possible reason for this is the poor telecommunications infrastructure of the Amazon and the non-existence of electricity in many rural areas of the Amazon.

### Analysis of Spatial Distribution

**Fig 5:**
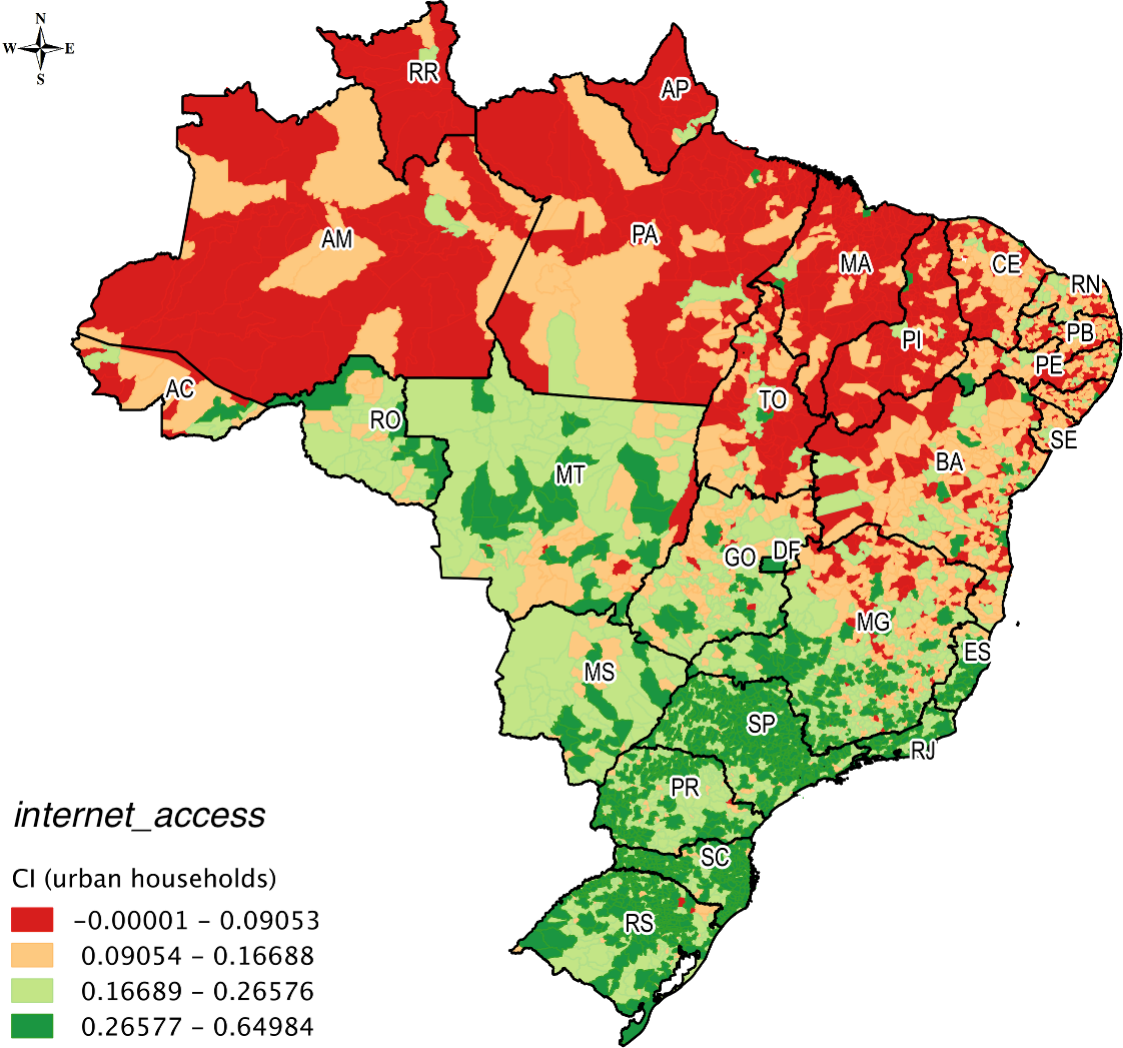
Spatial distribution of urban households in *internet_access*.

**Fig 6:**
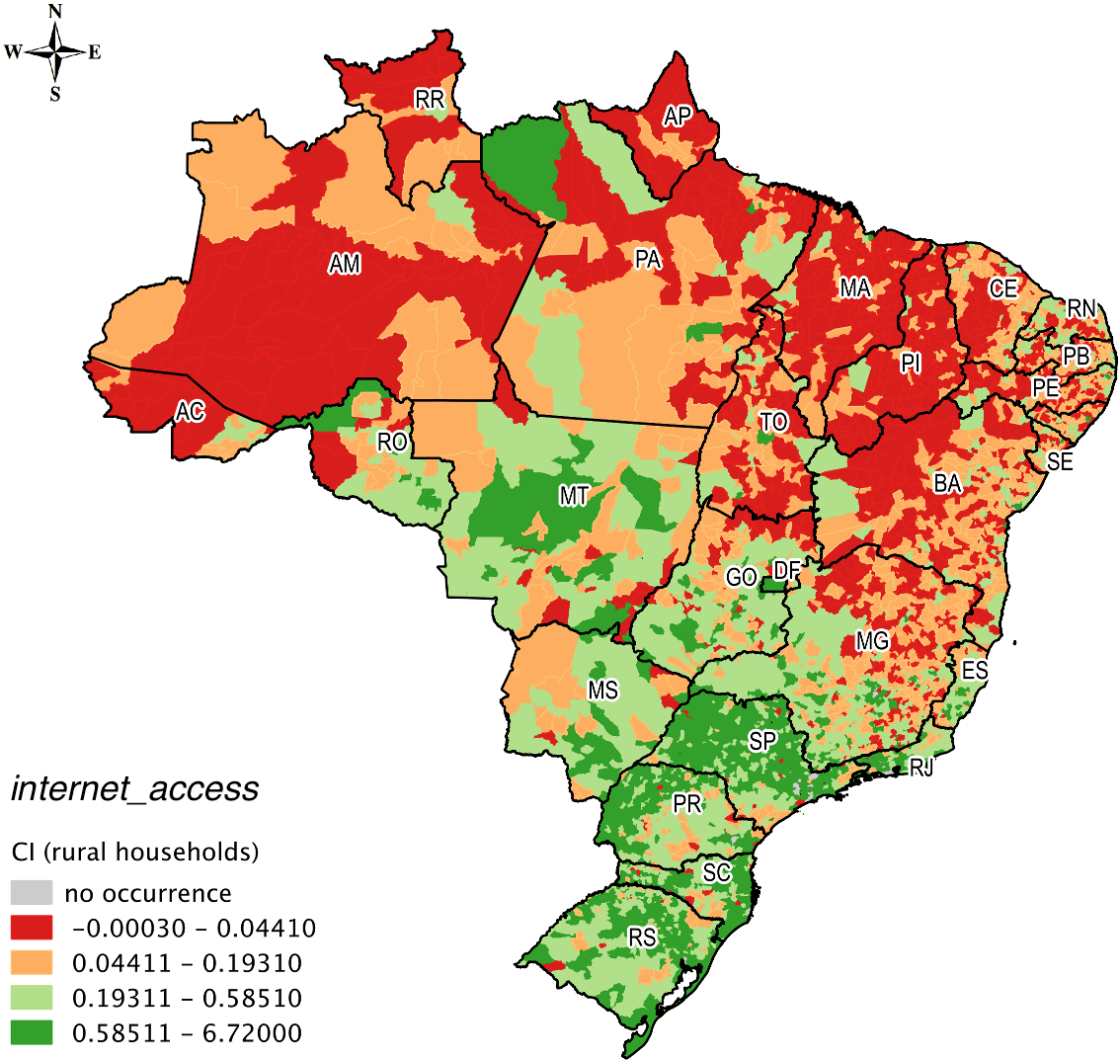
Spatial distribution of rural households in *internet_access*.

The spatial distribution of the concentration of urban and rural households for *internet_access* can be observed on the maps of Figs 5 and 6, respectively. The lowest ranges of CI values for *internet_access* for both urban and rural households were concentrated visibly in the Amazon and Northeast.

The municipality of Oriximiná in western Pará State had a very low concentration for urban households in *internet_access* (0.072) (Fig 5) and a high concentration for rural households (0.872) (Fig 6). This municipality, which is 818 km from Belém, the capital of Pará state, has the peculiarity of having an industry that extracts, beneficiates, and commercializes bauxite, which is the raw material for aluminum. This contributes to the development of rural areas in this municipality. In addition, Oriximiná has an advanced institute of higher education, research, and extension activities of the Federal Fluminense University (*Universidade Federal Fluminense*), which is a university headquartered in Rio de Janeiro state; this is an unusual situation in the Amazon. Furthermore, Parauapebas in southeastern Pará State had a very high concentration in *internet_access* (2.077) for rural households (Fig 6). This municipality, which is 719 km away from Belém, is situated in the largest mineral region on the planet, the Serra dos Carajás. In this municipality, the extraction of iron ore is the main source of income, which places the municipality among the biggest exporters by value (US$) of Brazil.

**Fig 7:**
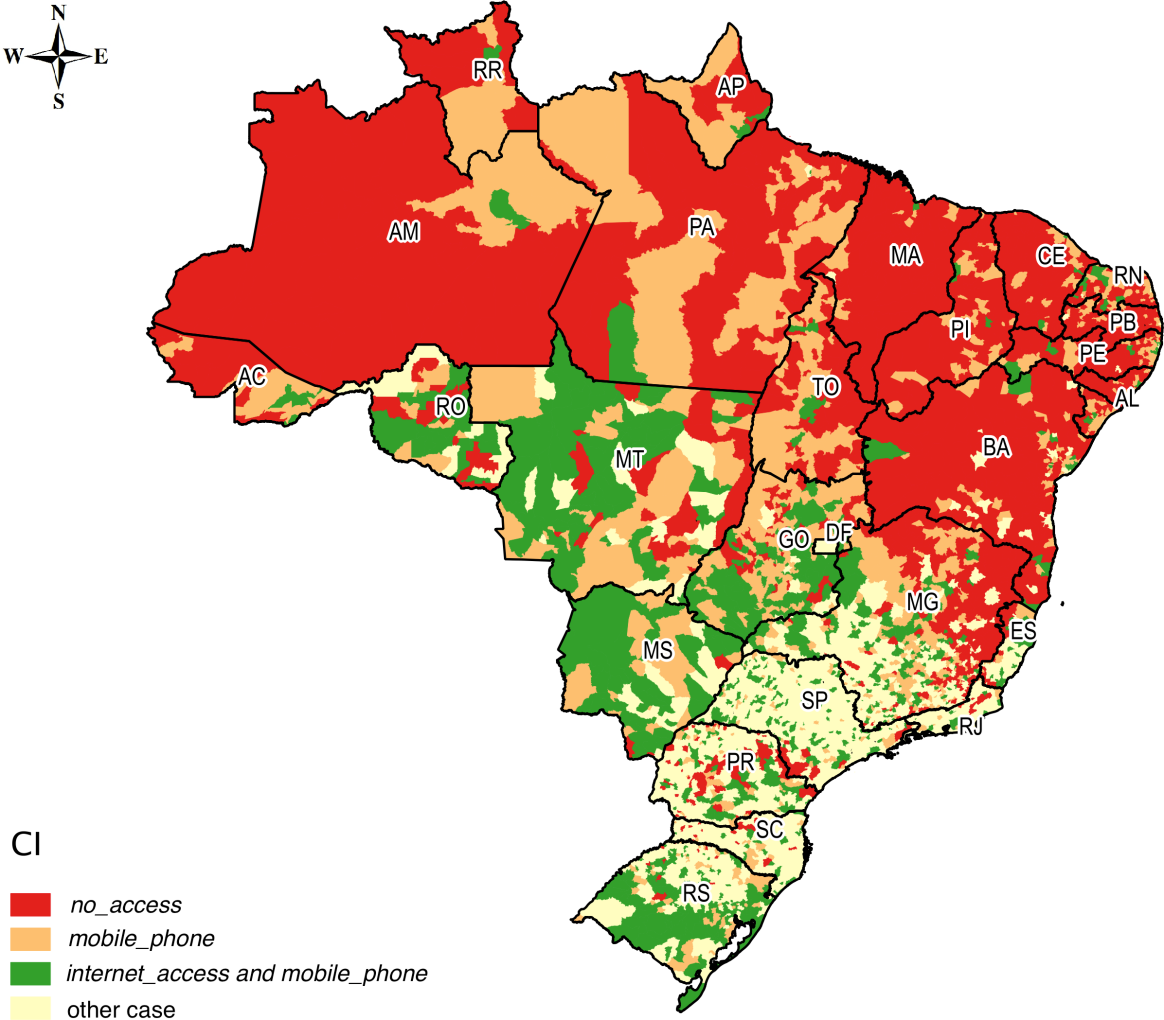
Spatial distribution of urban households in three different scenarios.

**Fig 8:**
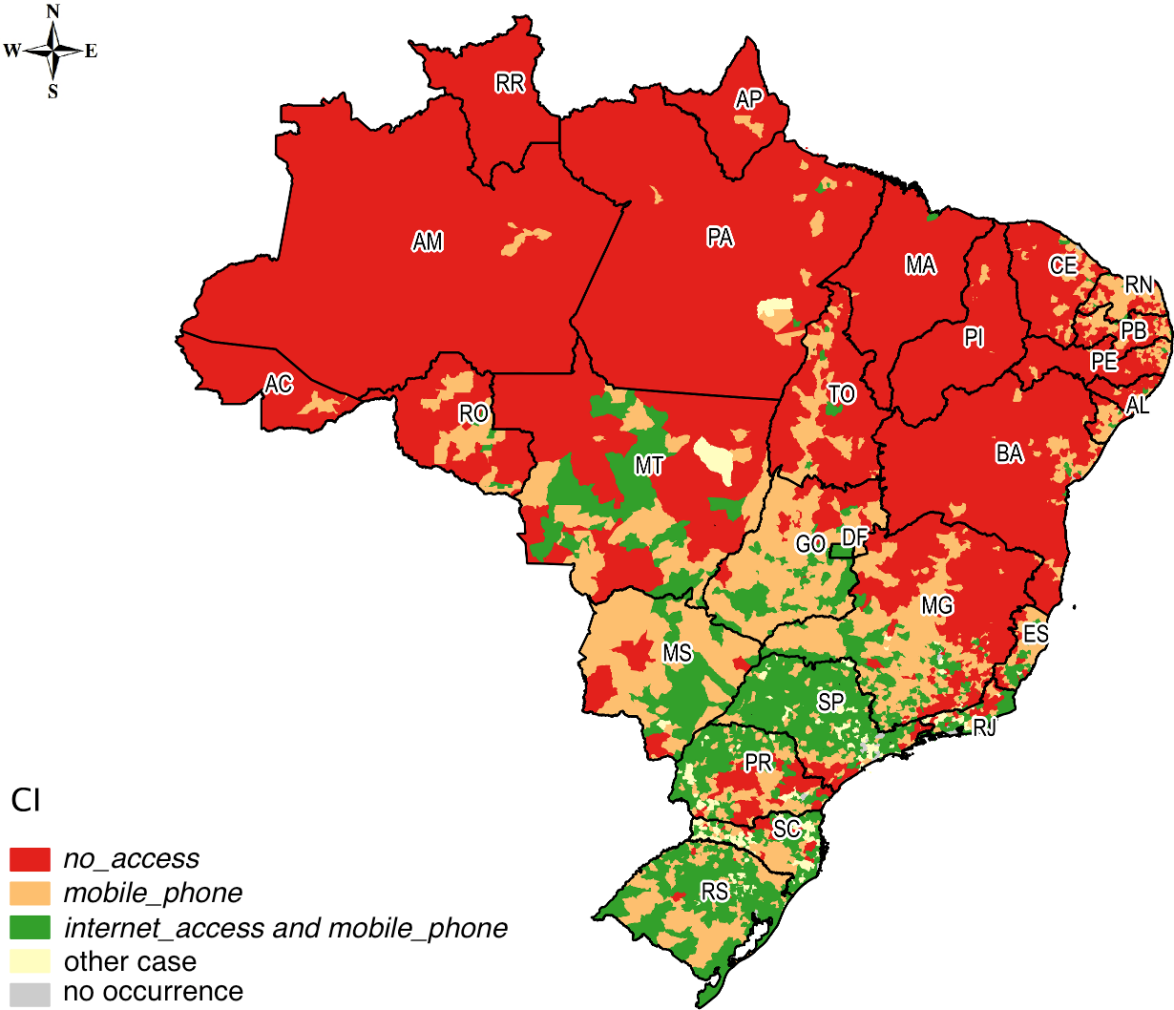
Spatial distribution of rural households in three different scenarios.

Using as a reference the average CI in Brazil for the studied classes, we selected the following scenarios of interest: (i) concentration in *no_access* above the average of the country; (ii) concentration in *mobile_phone* above the average of the country and below the average for *internet_access*, excluding the occurrences in (i); and (iii) concentration in *internet_access* above the average of the country, also excluding the occurrences in (i). For these scenarios, Figs 7 and 8 present the spatial distribution for urban and rural households, respectively.

Comparing Figs 7 and 8, we observe that the greater isolation scenario was concentrated in the rural Amazon and in the Northeast. These households were without computers with Internet access and without phones (fixed or mobile). On the other hand, the municipalities that had higher concentrations of *internet_access* and *mobile_phone* than the average of Brazil showed that agglomerations of households with better conditions of access to ICT were concentrated mainly in the Central-West, South, and Southeast of the country, in both urban and rural households. The concentration of households with above-average CI for *mobile_phone* and below average CI for *internet_access* reveals scenarios with potential for expanding access to the Internet.

### Search for Associations

Table 8 shows the RP for the existence of computers with Internet access, mobile phones, and fixed phones for urban and rural households, according to different classifications of municipalities: MHDI income, MHDI education, and municipality size. The processing results of the RP are accessible from the S3 File.

**Table 8:** RP in *internet_access*, *mobile_phone*, *fixed_phone*, *and no_access* According to Different Classifications of Municipalities.

Classification of municipalities	Number of municipalities	Urban and rural households			
		*internet_access*	*mobile_phone*	*fixed_phone*	*no_access*
**MHDI income**					
very high	51	0.353	0.143	0.276	0.051
high	1498	**0.489**	**0.444**	**0.485**	0.218
medium	2021	0.133	0.265	0.180	0.284
low	1804	0.024	0.142	0.056	**0.407**
very low	133	0.000	0.006	0.003	0.040
**MHDI education**					
very high	4	0.011	0.003	0.008	0.001
high	349	**0.544**	0.284	**0.476**	0.113
medium	1586	0.341	**0.384**	0.351	0.228
low	1993	0.084	0.218	0.117	0.304
very low	1575	0.020	0.111	0.048	**0.354**
**Municipality size**					
metropolis	18	0.362	0.194	0.286	0.081
large	264	**0.389**	0.321	**0.366**	0.174
medium	324	0.087	0.125	0.098	0.139
small	4901	0.162	**0.361**	0.250	**0.606**

Highest values are listed in bold.

Municipalities with high MHDI income were those with greater relative participation in *internet_access* (0.489), *mobile_phone* (0.444), and *fixed_phone* (0.485). In *internet_access*, the participation rate of municipalities with high or very high MHDI income was 0.842. On the other hand, municipalities with low or very low MHDI income were among 44.7% of households with *no_access*.

In addition, the *no_access* class was characterized by the highest relative participation of municipalities with low or very low MHDI education, and in this case, the participation rate was 65.8%. Municipalities with very low MHDI education were those with greater relative participation in *no_access* (0.354). Furthermore, high MHDI education was a feature of municipalities with greater RP in *internet_access* (0.544) and *fixed_phone* (0.476); in medium MHDI education, the participation rate in *mobile_phone* was 0.384.

With respect to population size, the common characteristic among municipalities that showed the highest RP in *mobile_phone* (0.361) *and no_access* (0.606) is small populations (less than 50,000 inhabitants). However, municipalities with populations of more than 100,000 inhabitants (metropolis and large municipality) were those with the highest RP in *internet_access*.

Among the 50 municipalities with the highest relative participation in *internet_access* for rural households, we found only 6 located in the Amazon: São José de Ribamar in Maranhão state, Barcarena and Parauapebas in Pará state, Sinop in Mato Grosso state, Porto Velho in Rondônia state, and São Luís in Maranhão state. On the other hand, for the 50 municipalities that contributed most to *no_access*, 30 were from the Amazon.

In addition, the presence of Amazon municipalities in the list of 50 municipalities with the highest RP for *household_internet* for urban households was low: we found only 4 such municipalities in the Amazon: Manaus in Amazonas state, Belém in Pará state, São Luís in Maranhão state, and Cuiabá in Mato Grosso state. Among the 50 municipalities with the highest RP in *internet_access* for urban households, all were either metropolises or large municipalities. The eight metropolises that contributed most to the RP were São Paulo, Rio de Janeiro, Brasília, Belo Horizonte, Salvador, Curitiba, Porto Alegre, and Fortaleza. None of these belongs to the Amazon. In addition, for *no_access*, the São Paulo, Rio de Janeiro, Fortaleza, Salvador, and Recife metropolises were those that contributed most, followed by Manaus and Belém, both located in the Amazon region.

As the RP take into account the percentage participation, it is natural that the metropolises tend to contribute more in all classes since they are the municipalities with the highest number of households. Thus for analysis, we selected the CI for urban households in all 18 metropolises, in descending order, for *internet_access* (S1 File). The first metropolis located in the Amazon (Belém) is in 13^th^ position. For the 11 Brazilian cities that have rural households, in descending order for *internet_access*, the three metropolises of the Amazon were among the four metropolises with lower concentrations of rural households with microcomputers with Internet (S2 File). For *no_access*, the Manaus and Belém metropolises occupied second and third positions with the highest CI, respectively.

The small municipalities are those that contributed most to *no_access* for urban and rural households: among the 50 with the highest CI for *no_access* among urban households, 26 were located in the Amazon (S1 File); for rural households, the scenario was even more critical because among the 50 with highest CI, 38 were located in the Amazon (S2 File).

The lack of basic infrastructure in smaller municipalities could arise from the geographic isolation of many of these municipalities [10, 24]. For example, only 1.1% of households in Autazes (AM), an isolated municipality surrounded by rivers in the Amazon rainforest, had *internet_access*; according Diniz et al. [10], this municipality was not served by banks until 2002.

**Fig 9:**
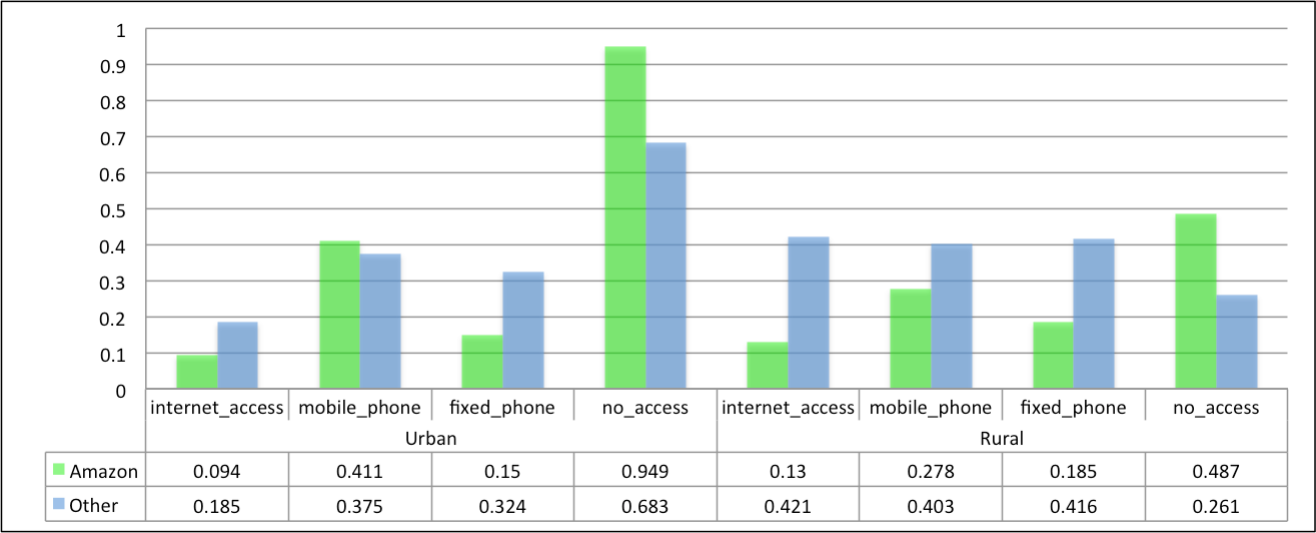
Average CI for Urban and Rural Households in Small Municipalities from the Amazon and Other Regions.

Fig 9 presents the average CI in all classes for urban and rural households in small cities in the Amazon in relation to other Brazilian regions. For *internet_access*, urban households located in the Amazon had a lower average concentration (0.094) than other regions of Brazil (0.185). For *no_access*, the average CI was higher in the Amazon (0.949 vs. 0683). Among rural households, the average CI in the Amazon displayed more significant differences compared to other regions. In particular, the average CI for *mobile_phone* was highest in the Amazon for urban households—justified by the low percentage of fixed phones in the households, owing to technical difficulties of installing infrastructure for fixed telephony services in the region.

In order to identify associations between parameters describing the ownership of ICT resources in urban and rural households and municipal indicators on income, education, electricity, and population size, we used Bayesian networks. Thus, for urban and rural households, we processed the *ict* for each municipality (S4 File). For both urban and for rural households, sorted in decreasing order by *ict*, there was a predominance of municipalities in the South and Southeast among the top 500 municipalities.

**Fig 10:**
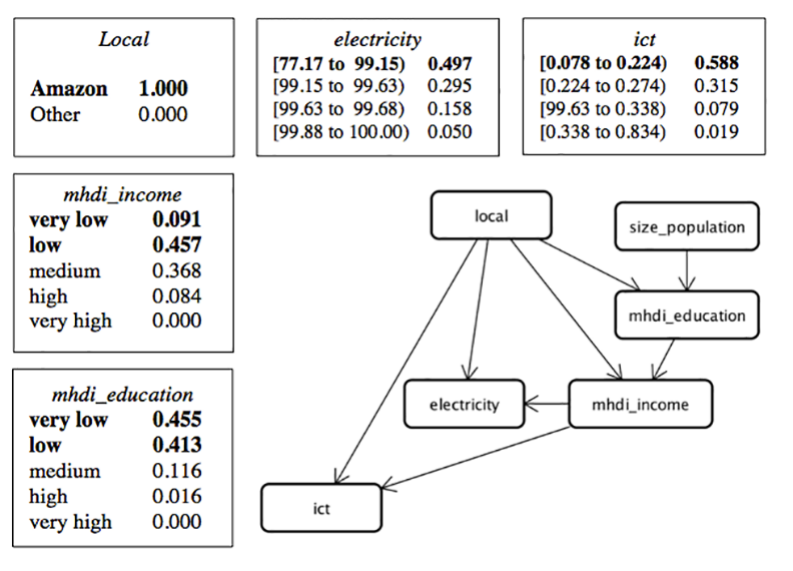
Structure of the Bayesian Network for Urban Households.

To set up the learning of the Bayesian network structure, we used the datasets composed of the variables *mhdi_education*, *mhdi_income*, *local*, *ict*, *electricity*, and *size_population* for urban households. Fig 10 presents the resultant Bayesian network for this dataset, according to values and ranges of values defined in Tables 2 and 3. For *electricity* and *ict*, the ranges of values were defined according to the frequency method.

For urban households, given the evidence that a municipality belongs to the Amazon region, the probability of this municipality having the lowest percentage of *electricity* is 0.497, having the lowest range of *ict* is 0.588, having *mhdi_income* = low or *mhdi_income* = very low is 0.548, and having *mhdi_education* = low or *mhdi_education* = very low is 0.868. However, when the evidence indicates that this municipality belongs to another region, the probability of this municipality having the lowest percentage of *electricity* falls to 0.208, having the lowest range of *ict* falls to 0.193, having *mhdi_income* = low or *mhdi_income* = very low is 0.340, and having *mhdi_education* = low or *mhdi_education* = very low is 0.613.

For municipalities with *mhdi_income* = high or *mhdi_income* = very high that do not belong to the Amazon, the probability that this municipality presents the highest range of *ict* for urban households is 0.591 and 0.862, respectively; for Amazon municipalities with *mhdi_income* = high (the Amazon has no municipalities with *mhdi_income* = very high), this probability falls to 0.033. Although *mhdi_income* is associated with ownership of ICT resources, other hypotheses can be considered to explain low *ict* in municipalities in the Amazon, such as their poor infrastructure for connectivity (fixed phone, mobile phone, and internet access), especially in smaller municipalities.

**Fig 11:**
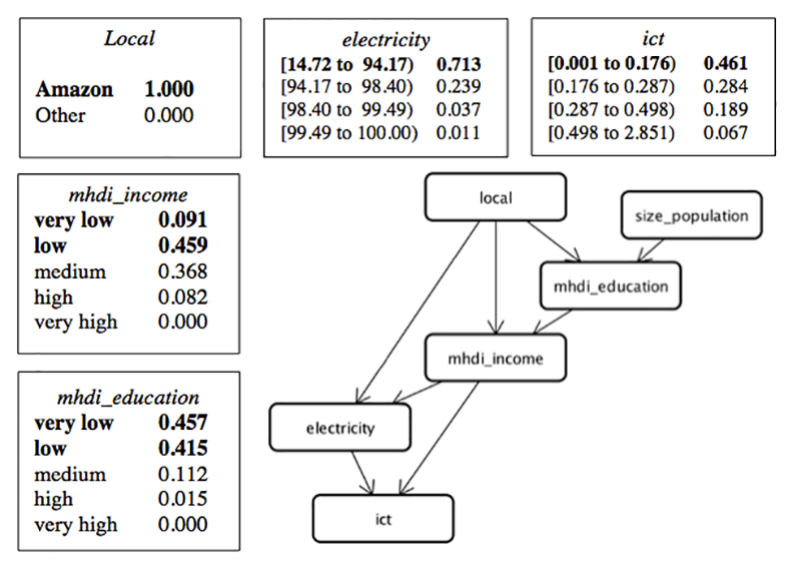
Structure of the Bayesian Network for Rural Households.

The same process was applied to rural households, resulting in the Bayesian network structure show in Fig 11.

In spite of the electricity network in the country reaching 98.73% of Brazilian households, for rural households in our study, the ownership of ICT resources showed a direct association with *electricity* (Fig 11), which does not occur with urban households (Fig 10).

When we observe rural households in the Amazon, the most frequent scenario involves municipalities with the lowest percentage of households with electricity (0.713) and lower ranges of *ict* (0.461), *mhdi_income* = low or *mhdi_income* = very low (0.550), and low *mhdi_education* = low or *mhdi_educatio*n = very low (0. 872). However, when the evidence indicates that the municipality belongs to another region, the probability of presenting the lowest percentage of electricity falls to 0.175, the lowest range of *ict* falls to 0.214, *mhdi_income* = low or *mhdi_income* = very low is 0.324, and *mhdi_education* = low or *mhdi_education* = very low is 0.619.

The association between MHDI income and access to ICT confirms the findings of other studies [17, 24–26, 29]. In addition, international statistics indicate the importance of considering the urban–rural digital divide [18, 20]. In our study, we address these issues and find, according to the Brazilian census, that the lack of energy—a challenge that has already been overcome in many urban areas of the country—proved to be a barrier for the ownership of ICT resources in rural areas.

## Conclusion

The results of the study of ICT concentration for Brazilian urban and rural household classes showed significant contrasts between municipalities of the Amazon compared to other regions of the country.

Our results showed high agglomerations of rural households without computers with Internet access, without mobile phones, or without fixed phones in the Amazon region compared to other regions of the country. These results confirm the arguments from the ITU [17] about the developing world: there is a growing rural–urban divide, since rural areas have lower 3G coverage, smaller proportions of households with Internet access, and fewer connected enterprises and schools compared with urban areas.

Even when the most recent data is observed from the Ministry for Communications on infrastructure investment for accessing the Internet in the municipalities (*Ministério das Comunicações do Brasil*; http://www.mc.gov.br/DSCOM/view/Principal.php), such as mobile broadband (3G Technology) coverage and the National Plan of Broadband, 187 municipalities in the Amazon did not have these services until November 2014. This applies to Santa Rosa do Purus in Acre state and the following municipalities, all located in Amazonas state: Itamarati, São Paulo de Olivença, Juruá, and Santa Isabel do Rio Negro.

A limitation of our study relates to the impossibility of conducting a time-series (historical) analysis of the association between variables. This is because the variable studied (*V0220*, households with computers with Internet access) was included only in the Demographic Census in 2010, when the last census was conducted. Another limitation is the lack of inclusion of a variable representing if households have computers without Internet access—we chose variables that represent the possession of equipment that allows bi- or multi-directional communication. However, we recognize that the ownership of computers without Internet access represents a valuable source of learning to deal with ICT.

For further research, we intend to include individual indicators (e.g., highest education level, ICT access, occupation, and location of Internet use) and indicators of municipalities (e.g., e-government services and the proportion of schools and public spaces with Internet access) in a similar study.

## Supporting Information

S1 File CI in *internet_access*, *mobile_phone*, *fixed_phone*, *and no_access* for Urban Households. (XLSX)

S2 File CI in *internet_access*, *mobile_phone*, *fixed_phone*, *and no_access* for Rural Households. (XLSX)

S3 File RP in *internet_access*, *mobile_phone*, *fixed_phone*, *and no_access* for Urban and Rural Households. (XLSX)

S4 File ICT index for Urban and Rural Households. (XLSX)
